# The mid-term outcomes of mobile bearing unicompartmental knee arthroplasty versus total knee arthroplasty in the same patient

**DOI:** 10.3389/fsurg.2023.1033830

**Published:** 2023-01-25

**Authors:** Jinlong Ma, Liang Zhang, Cui Wang, Kuishuai Xu, Zhongkai Ren, Tianrui Wang, Yingze Zhang, Xia Zhao, Tengbo Yu

**Affiliations:** ^1^ Department of Sports Medicine, The Affiliated Hospital of Qingdao University, Qingdao, China; ^2^ Department of Abdominal Ultrasound, Affiliated Hospital of Qingdao University, Qingdao, China; ^3^ Department of Traumatology, The Affiliated Hospital of Qingdao University, Qingdao, China

**Keywords:** unicompartmental knee arthroplasty, total knee arthroplasty, outcome, complication, revision

## Abstract

**Objective:**

To compare the mid-term outcomes of unicompartmental knee arthroplasty (UKA) that was performed in one knee and total knee arthroplasty (TKA) performed in the other knee in the same stage.

**Methods:**

This is a retrospective study. A total of 63 patients (126 knees) scheduled for one-stage knee surgery due to osteoarthritis of both knees were selected, and all patients underwent one-stage mobile platform UKA and TKA of the other knee. Differences in general clinical data, functional recovery, complications, and prosthesis revision rates were assessed after UKA and TKA, respectively. The evaluation indicators for knee joint function recovery included the hospital for special surgery knee score (HSS), Joint Forgotten Score (JFS), Knee Injury and Osteoarthritis Outcome Score (KOOS), and Visual analog scale (VAS). Patient preference between UKA and TKA was also recorded.

**Results:**

During a mean follow-up of 76.95 months (range, 65.00 to 87.00 months), there were no significant differences in postoperative complications between the two groups (*P* = 0.299); however, the prosthesis revision rate was higher in the UKA group than in the TKA group (*P* = 0.023). The incision length, operation time, blood loss, and postoperative drainage volume in the UKA group were significantly (*P* < 0.001) lower than those in the TKA group: JFS, ROM, and VAS in the UKA group were higher than those in the TKA group (*P* < 0.001, *P* = 0.023, *P* = 0.032), HSS and KOOS in TKA group were significantly (*P* < 0.001) higher than those in UKA group. At the last follow-up, 40% and 24% of patients preferred TKA and UKA, respectively.

**Conclusions:**

TKA was found to be superior to UKA in terms of HSS, KOOS, and VAS, while UKA had more significant advantages in terms of less surgical trauma, better ROM, and higher JFS. Complications were not different between groups, but UKA had a higher rate of prosthesis revision. After a follow-up of at least 5 years, more patients preferred TKA.

## Introduction

Total knee arthroplasty is an effective treatment for advanced knee osteoarthritis with pain associated with loss of function (1). Approximately one-third of patients experience knee-related symptoms, and approximately 20% require total knee arthroplasty (2). At the same time, differences were found in the location and degree of knee joint degeneration in some patients, where one side involved multiple compartments while the other involved unicompartmental degeneration. However, numerous studies investigated potential differences in the recovery of the knee joint after TKA combined with UKA in the treatment of knee arthritis, and the comparison of the clinical efficacy between the two remains controversial (3).

Previous studies have found some advantages of UKA over TKA, including less surgical trauma as well as preservation of the anterior cruciate ligament (4), with survival rates of 98% and 95% at 10 and 20 years, respectively (5). Simultaneous comparison of UKA and TKA revealed that UKA resulted in earlier recovery, less postoperative pain, and higher quality of life. Unicompartmental knee arthroplasty was associated with a better gait pattern compared to total knee arthroplasty (6), and patients reported feeling more like a normal knee after UKA (7, 8). Compared with TKA, UKA has fewer perioperative complications but a higher revision rate (3). After adjusting the preoperative flexion angle, UKA can achieve a postoperative flexion angle compared with TKA (8). UKA has been associated with better JFS and KOOS and is otherwise comparable to TKA, thus potentially presenting the preferable option (9). Yet, there are other studies with different findings, where UKA did not achieve good clinical efficacy 2 years after surgery (10).

Most previous studies have used a mutually controlled approach to eliminate the influence of demographic variables such as age, gender, and body mass index between the control and study groups. However, some factors, such as lifestyle, limb movements, and conditions for rehabilitation, are difficult to control and quantify. In addition, previous reports were limited by a small sample size, which we overcame in the present study by increasing the sample size. Also, the duration of follow-up time was more than 5 years for all patients.

In order to improve the study design, all patients included in this study underwent UKA in one knee, while TKA was performed in the other knee in one stage. We compared the mid-term clinical outcomes, complications, and revision rates after primary TKA with UKA in patients.

## Methods

### Study design and patients

This study is a retrospective study that evaluated KOA patients who received concurrent knee arthroplasty in the Affiliated Hospital of Qingdao University between June 2013 and August 2016. The Inclusion criteria were patients who underwent UKA in one knee and simultaneous TKA in the other knee. Exclusion criteria referred to patients with previous rheumatoid arthritis, history of knee arthritis infection or osteomyelitis, history of previous knee surgery, those lost to follow-up or who refused to join the experiment.

Our retrospective study was approved by the Ethics Institutional Review Board of the Affiliated Hospital of Qingdao University (Approval No.: QYFYWZLL26915), and all patients provided informed consent.

### Surgical methods and perioperative management

Both groups were operated by the same group of physicians, UKA was performed using Oxford III (mobile platform) prosthesis from Biomet, USA, and TKA was performed using Advance knee prosthesis (Wright, USA) or Scorpio NRG knee prostheses (Stryker, USA). All the procedures started with the left knee, and all patients were treated with conventional anterior midline knee incision and medial para-articular capsule incision. The patients in the UKA group were treated using the standard Oxfordphase3 minimally invasive unicompartmental knee arthroplasty operating manual. The TKA group was operated on according to conventional total knee arthroplasty. Anticoagulation was continued for 2 weeks after surgery in all patients, antibiotics were used to prevent infection for 24 h, and analgesia was continued until the patient's function returned to normal. The patients were started on partial weight-bearing and active assisted knee exercises and instructed to begin ankle pump exercises and quadriceps exercises as soon as possible. All patients underwent physical rehabilitation by the same rehabilitation technologist.

### Follow-up and data collection

A single assessor, who was not involved in the study design but was familiar with the assessment tools, was responsible for data collection, which was performed using standard case record forms. General clinical data included incision length, operation time, blood loss, and postoperative drainage volume. The knee function scores included: HSS (11), JFS (12), and KOOS (13). KOOS has 42 items that are divided across 5 categories, with 0∼4 points assigned for each item. The scores for each part of item are individually calculated and converted into a percentage system. Knee ROM was measured using a telescopic goniometer. The evaluation of pain was performed using a visual analog scale (VAS) score (14) ranging from 0 to 10, with 0 indicating no pain and 10 representing the maximum pain. We defined a complication as the presence of any of the following: lower extremity deep venous thrombosis, pulmonary embolism, infection, prosthesis loosening, periprosthetic fracture, and insert dislocation. Removal, replacement, or addition of any component of the prosthesis was considered as a revision, including exchange of the insert and change to TKA.

### Statistical analysis

All statistical analyses were performed using SPSS 25.0 software. Measurement data were expressed as mean and standard deviation (x ± s), and categorical data were expressed as numbers (percentage). The differences in the measurement data of patients in the UKA TKA group were analyzed using the independent sample t-test, and the differences in the categorical data between the two groups were analyzed using the chi-square test or Fisher's exact test. Revisions were analyzed using Kaplan-Meier survival curves. Test level *α *= 0. 05. A *P* value < 0.05 indicates statistical significance.

## Results

### Study flowchart, demographic characteristics, and the operation result

**Figure 1:**
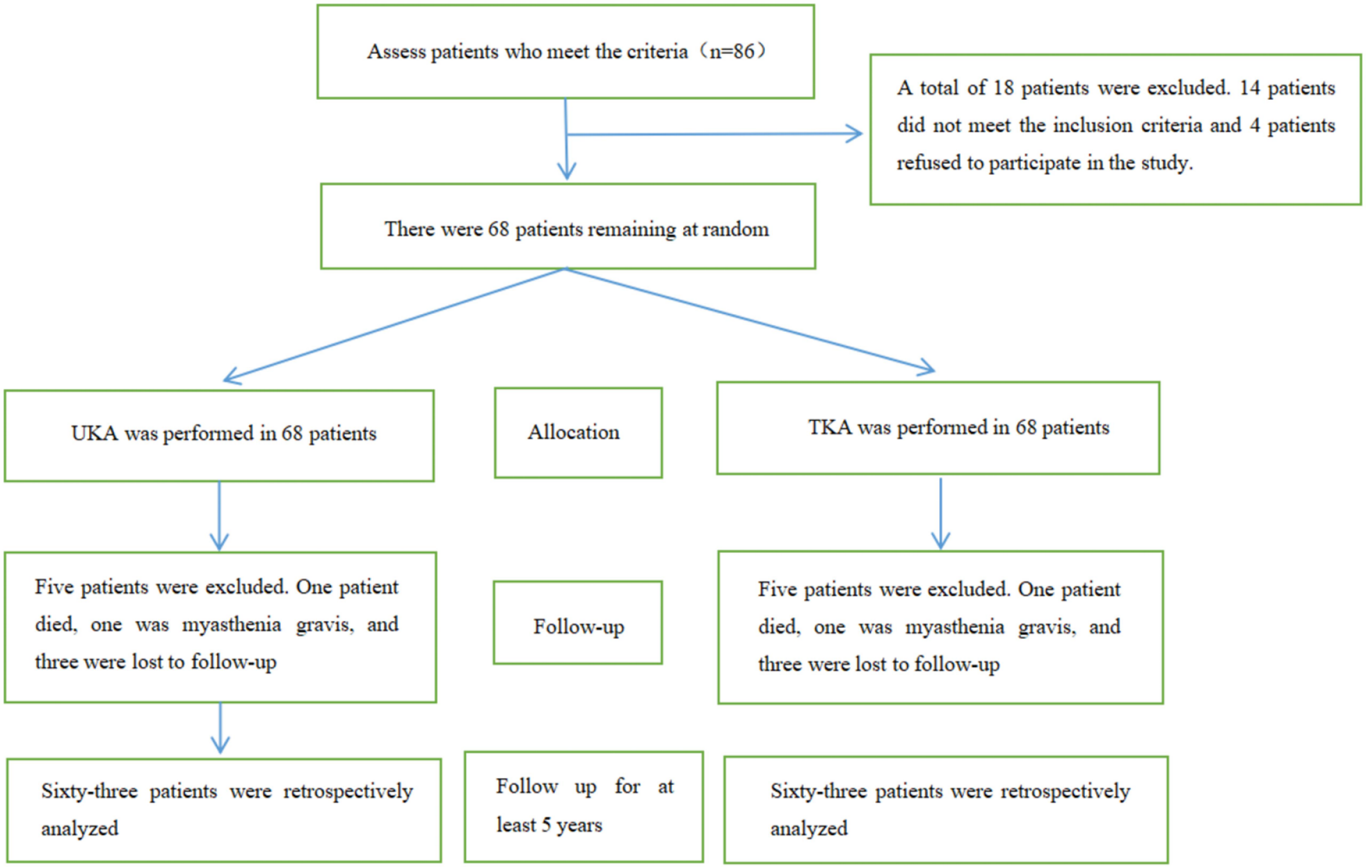
Study flowchart.

Among a total of 86 patients with degenerative arthritis who were assessed for possible inclusion in the study, 23 did not meet the inclusion criteria and thus were excluded. Finally, 63 patients (126 knees), 14 males and 49 females, with a mean age of 63.71 ± 6.65 years (range, 50∼80 years) and a mean BMI of 27.21 ± 2.98 kg/m^2^ (range, 20.9∼35.2 kg/m2) were included in the final analysis. The flowchart scheme of this study is shown in Figure 1; the baseline characteristics of all patients are summarized in Table 1; procedure-related outcomes for all patients are presented in Table 2.

**Table 1:** Demographic characteristics.

	Mean ± SD/*n* (%)	Range
***N* = 63**		
Age (years)	63.71 ± 6.65	50–80
**Gender, *n* (%)**		
Male	14 (22.22%)	-
Female	49 (77.78%)	-
BMI (kg/m^2^)	27.21 ± 2.98	20.1–35.2
**Pre-operative Kellgren-Lawrence classification**		
III	47 (37.3%)	-
IV	79 (62.7%)	-
Follow-up (months)	76.95 ± 5.72	65–87

BMI, body mass index.

**Table 2:** Intra-operative features.

	UKA (*N* = 63)	TKA (*N* = 63)	*t*	*P*
Length of the incision (cm)	11.3 ± 1.76	16.9 ± 2.12	−16.175	<0.001
Operative time (mins)	55.92 ± 11.31	64.97 ± 13.32	−4.109	<0.001
Blood loss (ml)	115.08 ± 42.15	223.02 ± 53.96	−12.512	<0.001
Postoperative drainage (ml)	188.41 ± 56.37	255.24 ± 62.24	−6.316	<0.001

UKA, Unicompartmental knee arthroplasty; TKA, Total knee arthroplasty.

### HSS, JFS, and KOOS

In the functional outcome, the mean JFS was significantly (*p* < 0.001) higher in the UKA group than in the TKA group, while the HSS and KOOS were significantly (*p* < 0.001) higher in the TKA group than in the UKA group (Table 3).

**Table 3:** HSS, JFS and KOOS scores in two groups.

		UKA (*N* = 63)	TKA (*N* = 63)	*t*	*P*
HSS	Preoperation	40.35 ± 5.74	38.78 ± 5.05	1.631	0.105
	Last follow-up	87.98 ± 4.36	90.89 ± 3.9	3.940	<0.001
**KOOS**					
	Preoperation	44.67 ± 6.75	39.73 ± 5.56	4.481	<0.001
	Last follow-up	90.25 ± 4.48	93.14 ± 2.6	−4.427	<0.001
JFS	Last follow-up	94.57 ± 2.58	88.1 ± 4.81	9.418	<0.001

UKA, Unicompartmental knee arthroplasty; TKA, Total knee arthroplasty; HSS, Hospital for special surgery knee score; JFS, Joint Forgotten Score; KOOS, Knee Injury and Osteoarthritis Outcome Score.

### ROM and VAS

The mean ROM was 124.06 ± 8.58 in the UKA group and 120.16 ± 10.43 in the TKA group, revealing a significant difference between the two groups (*p* = 0.023). The mean VAS was 1.89 ± 0.83 in the UKA group and 1.57 ± 0.82 in the TKA group, revealing a significant difference between the two groups (*P* = 0.032) (Table 4).

**Table 4:** ROM and VAS scores in two groups.

		UKA (*N* = 63)	TKA (*N* = 63)	*t*	*P*
ROM (°)	Preoperation	116.67 ± 9.89	113.89 ± 9.27	1.709	0.09
	Last follow-up	124.06 ± 8.58	120.16 ± 10.43	2.294	**0**.**023**
VAS					
	Preoperation	6.4 ± 0.55	6.52 ± 0.82	−1.018	0.311
	Last follow-up	1.89 ± 0.83	1.57 ± 0.82	2.169	**0**.**032**

UKA, Unicompartmental knee arthroplasty; TKA, Total knee arthroplasty; ROM, Range of motion; VAS, Visual analog scale.

### Complications

There was no significant difference in postoperative complications between the two groups at the last follow-up (*p* = 0.299) (Table 5).

**Table 5:** Local complications.

	UKA (*N* = 63)	TKA (*N* = 63)	*χ**^2^***	*P*
**Complications**				
Deep vein thrombosis	0	1		
Prosthetic joint infection	0	0		
Poor healing of the incisn	1	1		
Gasket dislocation	3	0		
Aseptic loosening	2	0		
Periprosthetic fracturs	0	0		
Stiffness	0	1		
Total	5	3	1.077	0.299

UKA, Unicompartmental knee arthroplasty; TKA, Total knee arthroplasty.

### Revision situation

**Figure 2:**
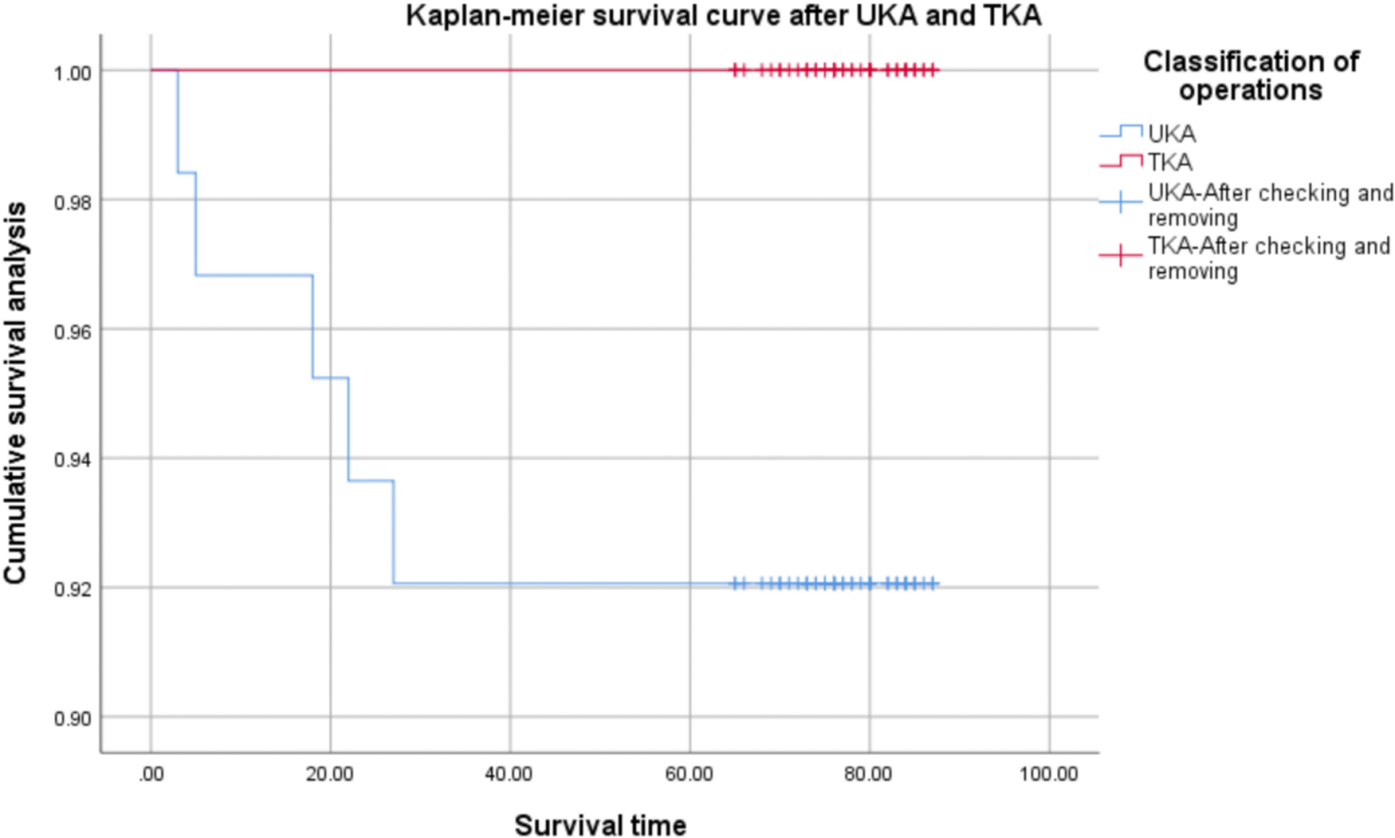
Survival curves of kaplan-Meier prosthesis in two groups.

The prosthesis survival rate was 100% during follow-up in the TKA group, and a total of 5 revisions occurred in the UKA group, with a prosthesis survival rate of 92.06%. The difference in prosthesis revision between the two groups was statistically significant (*P* = 0.023) (Table 6). From the Kaplan-Meier prosthesis survival curves of the two groups, it can be observed that the prosthesis survival rate in the TKA group was 100% and gradually decreased in the UKA group with a longer follow-up time (Figure 2).

**Table 6:** Comparison of knee prosthesis revision between two groups.

	UKA (*N* = 63)	TKA (*N* = 63)	*χ**^2^***	*P*
Revision for any reason, *n* (%)	5 (7.94%)	0	5.207	**0.023**

UKA, Unicompartmental knee arthroplasty; TKA, Total knee arthroplasty.

### Patient satisfaction

During follow-up, 15 (23.81%) patients chose UKA, and 25 (39.68%) patients chose TKA as a preferential approach, and 23 (36.51%) patients expressed no preference at least 5 years after surgery.

### Representative case

**Figure 3:**
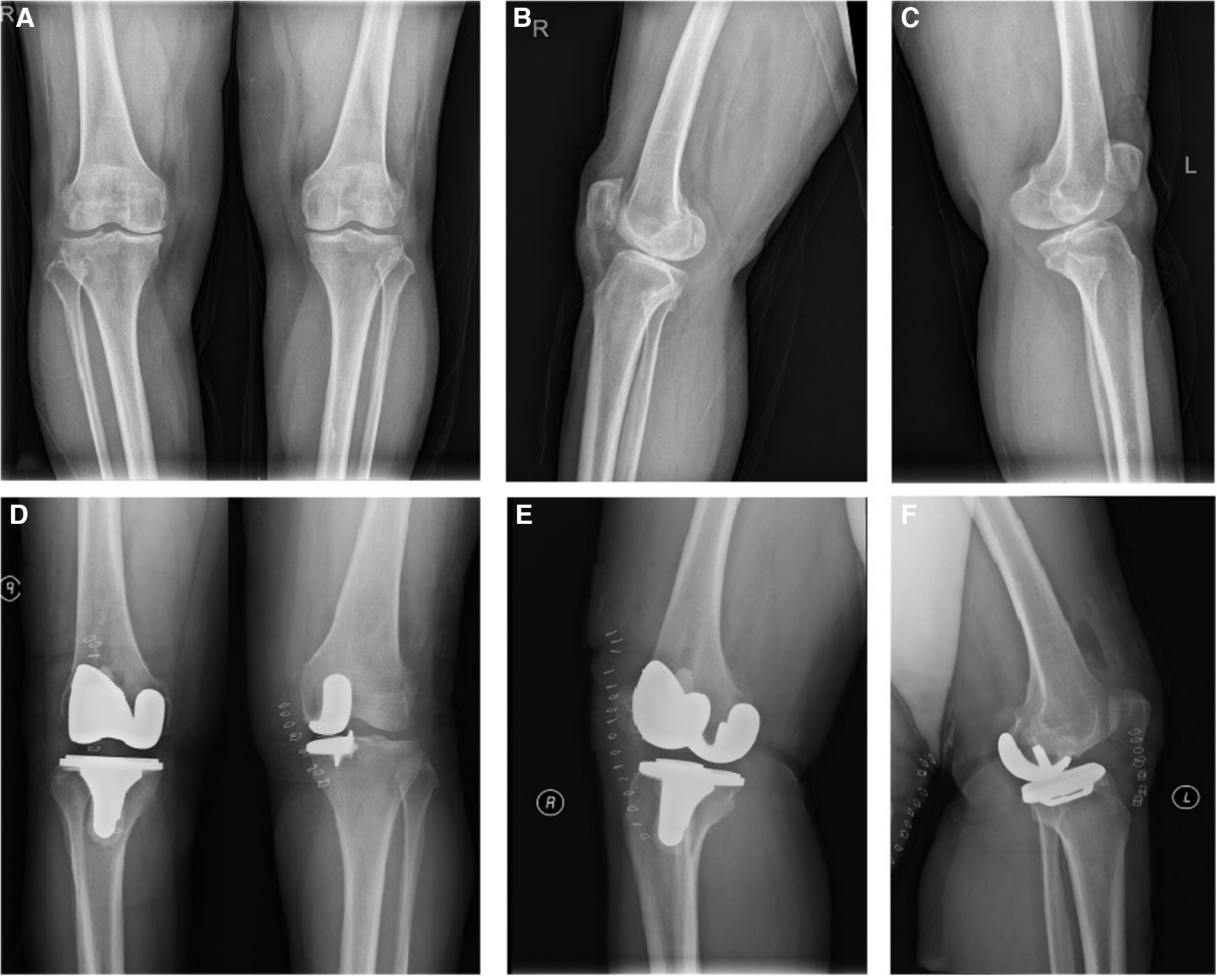
pre-operative (10 days) and post-operative (2 days) X-ray examinations of a 68-year-old women with bilateral osteoarthritis. (**A**) Anteroposterior position 10 days before operation. (**B**) Lateral position of right knee joint 10 days before operation. (**C**) Lateral position of left knee joint 10 days before operation. (**D**) Anteroposterior position 2 days after operation. (**E**) Lateral position of right knee joint 2 days after operation. (**F**) Lateral position of left knee joint 2 days after operation.

**Figure 4:**
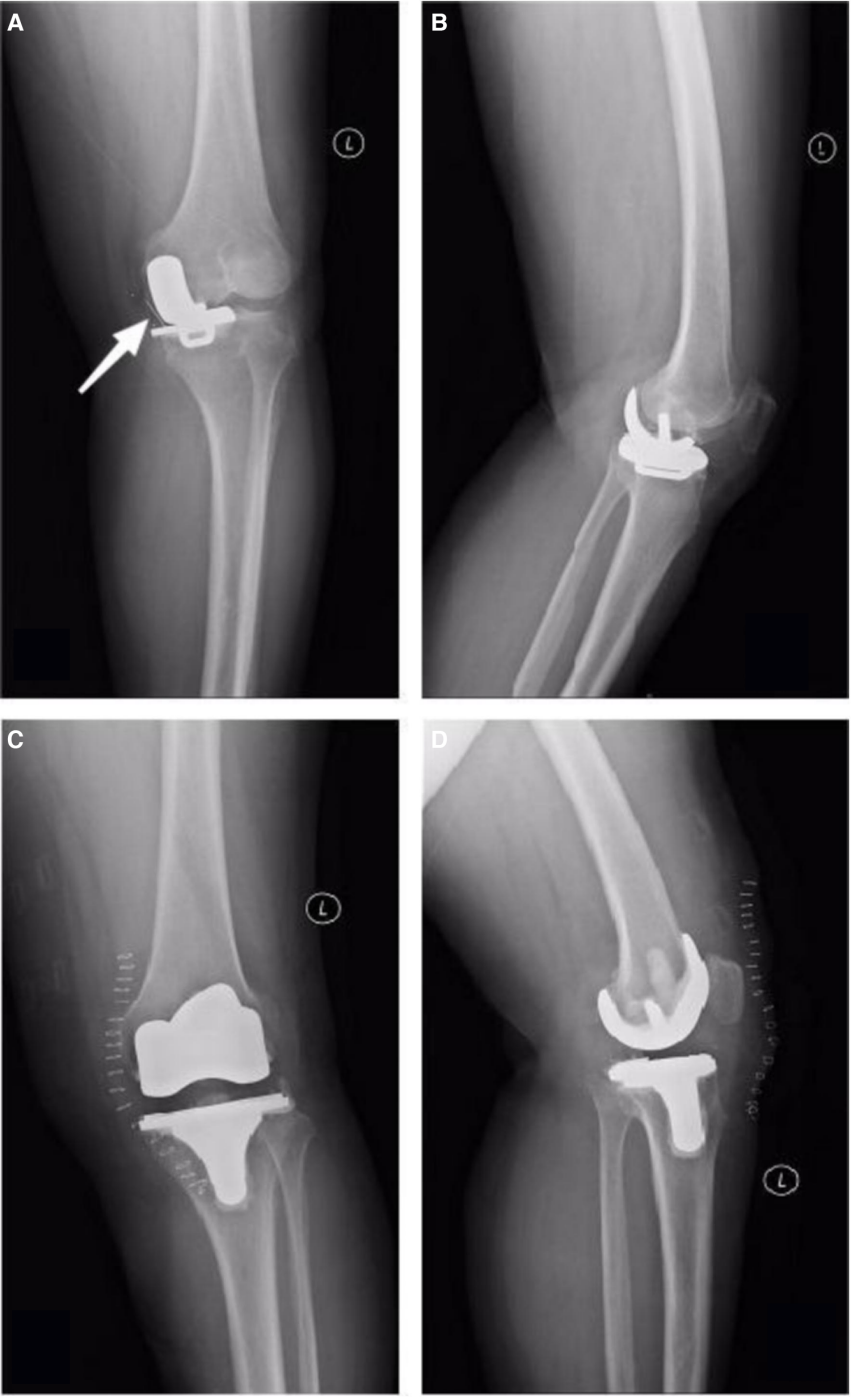
X-rays 5 months after UKA of the left knee and 3 days after conversion to TKA in a 65-year-old woman, White arrow: Poly backed out (**A**) Anteroposterior position of left knee joint 5 months after UKA, Poly backed out. (**B**) Lateral position of left knee joint 5 months after UKA. (**C**) The prosthesis of left knee joint was removed, and the surgical method was changed to TKA, anteroposterior position of left knee joint 3 days after TKA. (**D**) Lateral position of left knee joint 3 days after TKA.

A 65-year-old woman with osteoarthritis of the knee underwent UKA for the left knee and TKA for the right knee. Five months after surgery, a radiographic examination of the left knee revealed a dislodged polyethylene bearing, and the patient underwent removal of the left knee prosthesis and conversion to TKA, with good recovery after revision (Figures 3,4).

## Discussion

This retrospective clinical study compared UKA and TKA. All patients were followed up for at least 5 years. TKA had better clinical efficacy than UKA, while UKA had better ROM and JFS than TKA. There was no significant difference in complications between the two groups, but UKA had a higher rate of prosthesis revision. Also, more patients expressed their preference for the TKA approach.

Considering surgical results, UKA offers significant advantages in achieving smaller surgical incisions, reduced operative time, and lower intraoperative bleeding and drainage volume, which may lead to faster recovery, reduced morbidity, shorter hospital stays, and the need for rehabilitation. This conclusion is consistent with previous studies (15), which argued that UKA preserves the anterior cruciate ligament, preserves femoral and tibial bone reserves, reduces intraoperative and postoperative blood loss, shortens hospital stay and preserves normal biomechanics of the knee joint compared to TKA. Then again, we compared UKA and TKA clinical results, finding some differences between the two surgical modalities, with HSS and KOOS being significantly (*p* < 0.001) higher in the TKA group than in the UKA group. In their study, Boonchana et al*.* (9) found that UKA was associated with better JFS and KOOS and was otherwise comparable to TKA. In a previous comparison of TKA and UKA, the function was assessed according to the HSS and function scores, revealing the same efficacy between the two groups (16). Furthermore, Dalury et al*.* also found that the knee social score increased from 45.9 to 89.7 for UKA and from 42.4 to 90.3 for TKA, with little or no difference in results between the two procedures (17).

Considering subjective preference, 15 (23.81%) patients chose UKA, 25 (39.68%) patients chose TKA, and 23 (36.51%) patients had no preference after at least 5 years after surgery, which was not consistent with previous studies. According to existing literature, the vast majority of patients prefer UKA because of shorter hospital stays and faster rehabilitation (15). In the present study, patients were followed up for 5 years or more after surgery, and the subjective preference of the patients was directed more towards the assessment of clinical efficacy and pain, which is one of the reasons for the inconsistency with previous studies. However, we did not include studies for systematic assessment of patient satisfaction, which is also a limitation of the present study. Other studies reported that of 23 patients, 11 knees had no preference, 12 preferred UKA, and none preferred TKA (16). Dalury et al. (17) conducted a similar study that included 23 patients who underwent surgery, none of whom expressed preferred TKA at a mean follow-up of 42 months. In another similar study, 11 patients preferred UKA over TKA, 3 preferred TKA, while 9 said they felt the same about the two approaches (18). One of the possible reasons that patients in previous studies preferred UKA might be shorter hospital stays and greater postoperative range of motion. However, that study had a relatively shorter follow-up, with significantly more knee pain and higher revision rates in UKA and significantly less pain after TKA in patients at longer postoperative follow-up, which may be related to wear of the lateral cartilage in some patients in UKA. In the present study, we found that more patients preferred TKA.

A previous study showed that UKA had better ROM, and there was no difference in preoperative and postoperative pain (*P* > 0.05) (17). Takafumi et al*.* (8) designed a more precise study, and after adjusting for the preoperative flexion angle, UKA could achieve the postoperative flexion angle compared with TKA. In our study, the ROM of the UKA group was higher than that of the TKA group, which is consistent with previous literature suggesting that UKA provides higher ROM, lower incidence of stiffness, and lower need for rehabilitation as it preserves the anterior cruciate ligament and maintains normal knee structure (19, 20). In addition, we found that patients in the UKA group had a higher VAS than those in the TKA group. This is not consistent with previous studies (17, 18) and may be related to asymmetric rehabilitation exercises of the knee joint, asynchronous recovery of lower extremity function, or the revision rate and wear of the lateral compartment in the UKA group. Further follow-up observations are needed to establish whether the VAS will change with a longer follow-up time.

There was no difference between the two groups in terms of complications, which is consistent with the results of previous studies (16–18). However, we found that the survival rate of the prosthesis was 100% in the TKA group and only 92.06% in the UKA group during follow-up. As shown by the Kaplan-Meier prosthesis survival curve of the two groups, the survival rate of prosthesis in the UKA group gradually decreased with the increase in follow-up time. The main reason for the revision was the dislocation of the insert (60%). The used polyethylene tibial design may lead to a higher failure rate. Previous studies have also revealed that the survival rate in the UKA group was 85%, while that of the TKA group was 100%. These results suggest that UKA may not provide similar survival rates compared with TKA (16). In their study, Saenz et al. (21) reported on 113 patients (144 knees) who underwent UKA with an all-polyethylene tibial component and observed an implant survival rate of 89% at a mean follow-up of 36 months. Another study that used the Swedish registry evaluated 8,793 UKAs performed between 1998 and 2007, where 24% of UKAs were revised compared with 9% of all TKAs performed in the same time period (22). A study in 2017 reached a different conclusion, retrospectively analyzing all patients aged 75 years and older who were treated with UKA or TKA between 2002 and 2012, revealing no significant difference between UKA and TKA in the comparison of 5-year prosthesis survival (23). In the past 20 years, the increased survival rate of UKA may be related to the design of the prosthesis and the improvement of surgical techniques. In addition, since the requirement of UKA may be very high, the inexperience of surgeons may lead to the incidence of technical errors and early failure.

The present study has some limitations: (1) it is a single-center clinical trial, and other institutions should conduct similar surveys to confirm the reported results; (2) due to the limited number of patients suitable for simultaneous knee replacement surgery, the number of patients is still relatively small; (3) in this work, only preoperative and follow-up investigation studies were performed; the differences in the early stage of surgery were not compared, and no continuous dynamic follow-up was performed; (4) only HSS, JFS, and KOOS were compared, while other clinical scores were not considered. Despite these limitations, we compared the same patients and followed them for at least 5 years, thus increasing the credibility of the presented results.

## Conclusion

By comparing UKA with TKA, we found that TKA was superior to UKA in terms of HSS, KOOS, and VAS, while UKA had more significant advantages in obtaining less surgical trauma and achieving better ROM and higher JFS. Complications were not different between groups, but UKA had a higher rate of prosthesis revision. After a follow-up of at least 5 years, more patients preferred TKA.

## Data Availability

The raw data supporting the conclusions of this article will be made available by the authors, without undue reservation.
